# The ‘over‐the‐top’ technique allows for accurate and reproducible restoration of the native tibial slope in kinematically aligned total knee arthroplasty: A retrospective comparative analysis from the FP‐UCBM Knee Study Group

**DOI:** 10.1002/ksa.12750

**Published:** 2025-07-07

**Authors:** Edoardo Franceschetti, Giancarlo Giurazza, Stefano Campi, Stephen M. Howell, Alexander J. Nedopil, Giuseppe Francesco Papalia, Pietro Gregori, Biagio Zampogna, Rocco Papalia

**Affiliations:** ^1^ Fondazione Policlinico Universitario Campus Bio‐Medico Roma Italy; ^2^ Department of Medicine and Surgery Research Unit of Orthopaedic and Trauma Surgery, Università Campus Bio‐Medico di Roma Roma Italy; ^3^ Department of Biomedical Engineering University of California Davis California USA; ^4^ Department of Orthopaedic Surgery König‐Ludwig‐Haus University of Würzburg Würzburg Germany

**Keywords:** kinematically aligned total knee arthroplasty, over‐the‐top technique, posterior tibial slope, sagittal alignment, tibial component positioning

## Abstract

**Purpose:**

A critical challenge in kinematically aligned total knee arthroplasty (KA TKA) is achieving accurate sagittal alignment of the tibial component. Correct reproduction of the native posterior tibial slope (PTS) is essential for proper posterior cruciate ligament (PCL) tensioning, minimising wear of the polyethylene insert while ensuring stability on dense subchondral bone. This study assessed the accuracy of manually setting the tibial slope in KA TKA, comparing the conventional technique with the novel 'over‐the‐top' technique, hypothesising that the latter would provide greater accuracy and reproducibility, even for less experienced surgeons.

**Methods:**

A retrospective analysis was performed on 81 patients (90 knees, 29 operated by a senior resident) using the conventional KA technique and 83 patients (90 knees, 32 operated by a senior resident) using the ‘over‐the‐top’ technique. The latter involves using an angel wing placed through the cutting slot of the tibial guide and over the anterior and posterior rims of the medial tibial plateau to set the PTS. Pre‐ and postoperative PTS were measured on lateral radiographs, and the PTS difference (PTSD) was calculated. Patients were classified as inliers (PTSD ≤ ±2°) or outliers (PTSD > ± 2°).

**Results:**

Mean pre‐ and post‐operative PTS were 5.7 ± 3° and 6.4 ± 3.1° in the control group, and 5.6 ± 2.9° and 5.3 ± 2.9° in the study group. The study group had significantly lower mean PTSD (−0.3° ± 0.7°) compared to the control group (0.7° ± 2°; *p* < 0.001), with 0% and 25.5% outliers, respectively. Tibial recuts were required in 0% of cases in the study group and 15.6% in the control group (*p* < 0.0001), with differences between the lead surgeon (9.8%) and senior resident (27.6%).

**Conclusion:**

The ‘over‐the‐top’ technique provides superior accuracy and reproducibility in restoring the native PTS compared to the conventional KA technique and can be safely adopted even by less experienced surgeons.

**Level of Evidence:**

Level III, retrospective comparative study.

**Abbreviations**
ICC: intraclass correlation coefficient
KA: kinematic alignment
MA: mechanical alignment
PACS: picture‐archiving communication system
PCL: posterior cruciate ligament
PROMs: patient‐reported outcome measures
PTS: posterior tibial slope
PTSD: posterior tibial slope difference
TKA: total knee arthroplasty
UKA: unicompartmental knee arthroplasty

## INTRODUCTION

Despite the current focus on the coronal alignment of prosthetic components [8, 14, 18], a critical challenge in kinematically aligned total knee arthroplasty (KA TKA) remains the accurate sagittal alignment of the tibial implant. Unlike mechanical alignment (MA), which typically targets a fixed slope (e.g., 0°–3° or 5°–7°), depending on prosthesis design and posterior cruciate ligament (PCL) integrity, KA aims to restore a patient's native posterior tibial slope (PTS), which can vary widely, up to 20° [6, 16, 24, 29].

Accurate reproduction of the slope is essential for proper tensioning of the PCL and to prevent excessive stress and wear on the posterior aspect of the polyethylene insert, while ensuring stable placement on dense subchondral bone [19, 27]. Errors in slope adjustment continue to be a leadingcause of failure in KA TKA [27], which manifest as posterior subsidence and overload of the posterior portion of the polyethylene insert.

This study aimed to assess the accuracy of manually setting the tibial slope in KA TKA, comparing the conventional KA technique [26] with the new 'over‐the‐top' technique. The hypothesis was that the latter would be more accurate in establishing the tibial cut orientation in the sagittal plane and more reproducible, even by less experienced surgeons.

## MATERIALS AND METHODS

Institutional review board approval was granted for this research (IRB No. 32.19 OSS) and all participants provided informed consent. No financial incentives were provided for participation. A retrospective analysis of prospectively collected data from the FP‐UCBM Database was performed. Patients with knee osteoarthritis classified as stage IV according to the Kellgren–Lawrence classification, who underwent unrestricted KA TKA at our Institution using either the ‘over‐the‐top’ or the 'conventional' [26] KA technique, were deemed eligible for inclusion. The exclusion criteria included unavailability of preoperative and/or 6‐week postoperative true lateral radiographs, poor image quality and rotation, PCL insufficiency or PCL iatrogenic lesions.

In the study group, three patients were excluded due to poor‐quality postoperative radiographs and one due to PCL insufficiency, resulting in 83 consecutive patients (90 knees) who underwent KA TKA using the ‘over‐the‐top’ technique between February and May 2024.

In the control group, two patients were excluded due to poor‐quality postoperative radiographs, and two for PCL insufficiency, resulting in 81 consecutive patients (90 knees) who underwent KA TKA using the 'conventional' technique between November 2023 and January 2024, immediately prior to the implementation of the new technique.

A post hoc power analysis was conducted using G*Power 3.1 to assess the study's statistical power. With two equal groups of 90 knees each, an *α* level of 0.05, and an effect size (*d*) of 0.667, the power (1–*β*) was calculated to be 99.2%.

### Operative technique

All patients underwent calipered KA TKA using a standard medial parapatellar approach. To account for variability in cartilage thickness on the unworn side [12], and to ensure accurate restoration of the varus‐valgus and internal‐external rotation of the femoral component, residual cartilage thickness was systematically measured with a previously validated method [11]. In case of cartilage thickness greater than 2 mm, it was removed by both distal and posterior femoral condyles and bone resections were performed using a 2 mm spacer on both sides, with tolerance of ±0.5 mm to the implant thickness.

**Figure 1:**
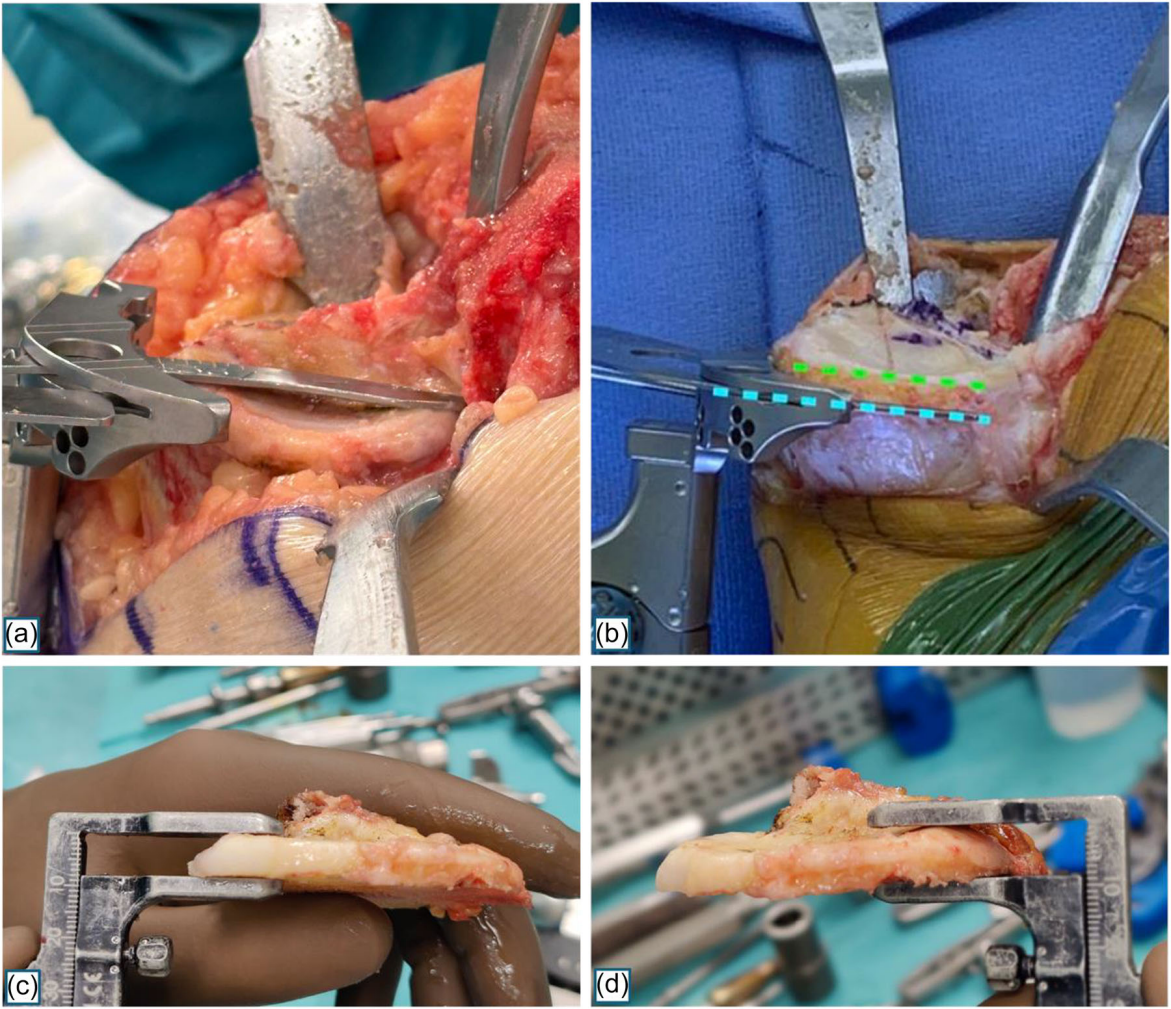
Over‐the‐top technique (a) versus conventional technique (b). Caliper measurements of the tibial cut at the level of the posterior (c) and anterior lip (d).

In the study group, the orientation of the tibial cut in the sagittal plane was established using the novel ‘over‐the‐top’ technique, which involves the following steps (Figure 1a):

‐ PCL is assessed both visually and with posterior drawer test to confirm its competence.
‐ The tibia is subluxated anteriorly with the aid of a Hohmann retractor, achieving complete exposure of both the anterior and posterior rims of the medial tibial plateau.
‐ Rotation and coronal alignment of the tibial cut are set using the long axis of the lateral plateau's oval and with two styli positioned at the base of the tibial spines, respectively.
‐ An angel wing is inserted into the cutting slot of the tibial guide and placed ‘over‐the‐top’ of the medial tibial plateau.
‐ The slope is adjusted until the angel wing touches both the anterior and posterior rims of the medial tibial plateau.
‐ The angel wing is then removed, and final height is set before proceeding with the saw blade.

In the control group, the conventional KA technique was applied, differing only in the positioning of the angle wing. Inserted through the cutting slot of the tibial guide, it is placed along the medial border of the medial tibial plateau (Figure 1b). The slope is then set solely by visual assessment, adjusting the plane of the angel wing to be parallel to the posterior slope of the medial joint line [26].

In both groups, following completion of the tibial cut, the slope of the tibial biscuit was meticulously verified by both visual inspection and using a caliper to measure the thickness at the level of the anterior and posterior rims (Figure 1c,d). Since manually adjusting the extramedullary tibial cutting guide with accuracy finer than 1 mm would be virtually impossible, errors of this magnitude were considered negligible. Therefore, only differences exceeding 1 mm were deemed significant for consideration of a tibial recut.

A targeted 2:1 allocation between the lead surgeon (E.F.) and senior resident (G.G.) was implemented as part of our Institution's training programme, resulting in 58 and 32 cases in the study group and 61 and 29 cases in the control group, respectively.

### Radiographic analysis

**Figure 2:**
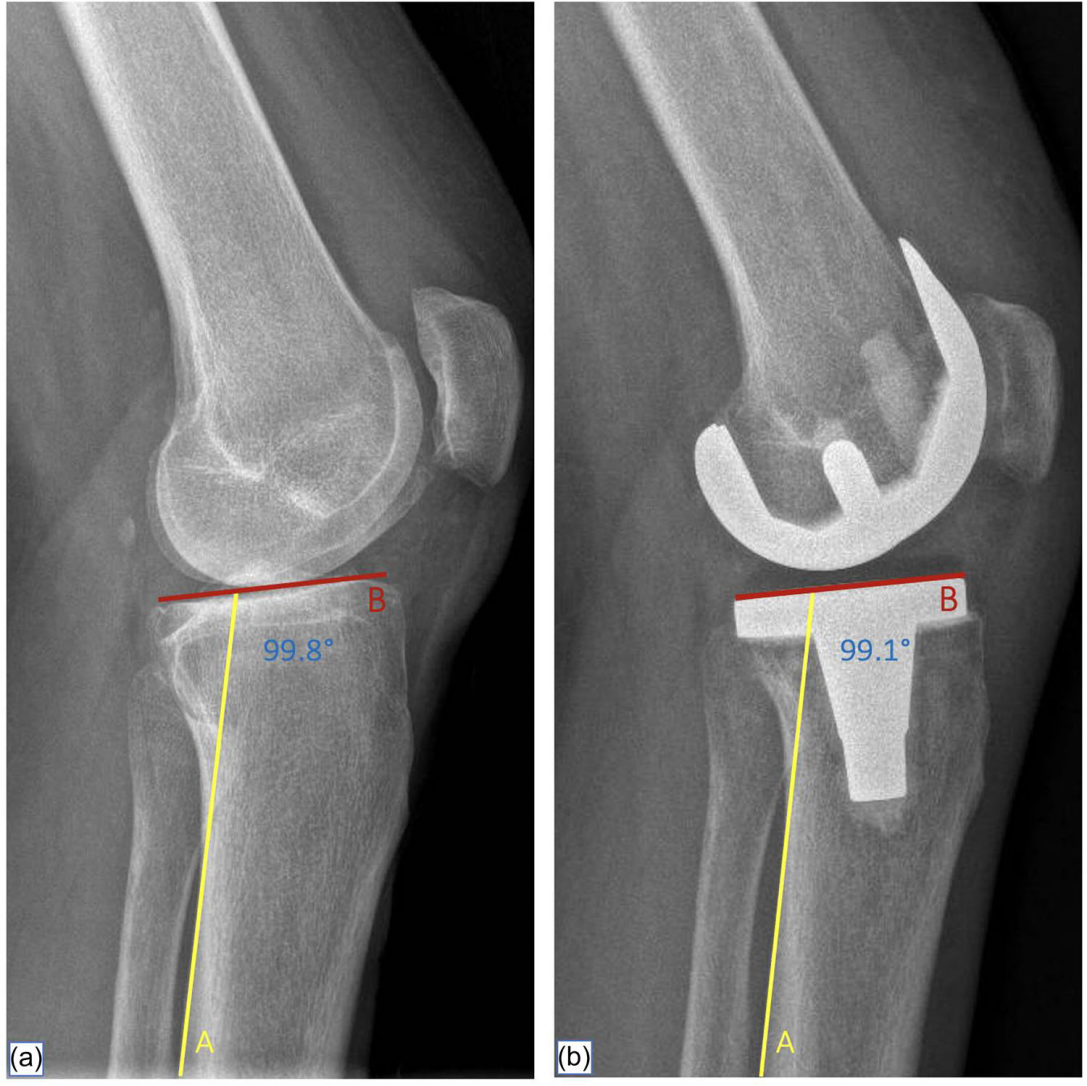
Posterior tibial slope difference (PTSD). (a) Native medial tibial plateau sagittal slope. Line A: posterior cortical line; Line B: line connecting the anterior and posterior edges of the medial tibial plateau. (b) Prosthetic sagittal slope. Line A: posterior cortical line; Line B: line parallel to the superior border of the tibial component.

For both the study and the control groups, native medial PTS and prosthetic PTS were measured on pre‐operative and 6‐week post‐operative standard true lateral standing radiographs, using the posterior cortical diaphyseal line as a vertical reference [4, 7]. Strict exclusion criteria were applied to minimise issues related to poor tibial rotation and variations in tibial shaft length included in the image. Specifically, only true lateral radiographs with overlapping posterior femoral condyles, a centred knee joint, and capturing at least 15 cm of the proximal tibia were deemed adequate [5]. Measurements were conducted using tools available in a picture‐archiving communication system (PACS) and recorded to the nearest 0.1°. The difference between prosthetic and native PTS was defined as Posterior Tibial Slope Difference (PTSD) (Figure 2). Positive values denoted an increase and negative values a decrease in the prosthetic slope compared to the native slope.

Based on these measurements rounded to the nearest 0.5°, the patients were divided into two groups [26]:

‐ Inliers: PTSD ≤ ± 2°
‐ Outliers: PTSD > ± 2°.

### 2.3 Statistical analysis

The Excel® and Power Query® software were employed for descriptive data analysis. Continuous variables were described using means, standard deviation, and ranges, while categorical variables were expressed as percentages. Statistical analysis was performed using SPSS (version 26.0; SPSS Inc.) and statistical significance was defined as *p* < 0.05.

Radiographic measurements were performed on each subject twice, 6 weeks apart by one of the authors (P.G.) who was blinded to the patient's allocation to either the study or control group and the average of these two values was recorded. Furthermore, the same measurements were performed once in 20 subjects by two authors (P.G. and B.Z.). The intraclass correlation coefficient (ICC) was utilised to assess the intra‐observer and inter‐observer reliability of radiographic measurements. The obtained ICC values indicated excellent [13] reliability in both intra‐observer (0.97; 95% CI, 0.961–0.975), and inter‐observer assessments (0.94; 95% CI, 0.938–0.957).

## RESULTS

**Figure 3:**
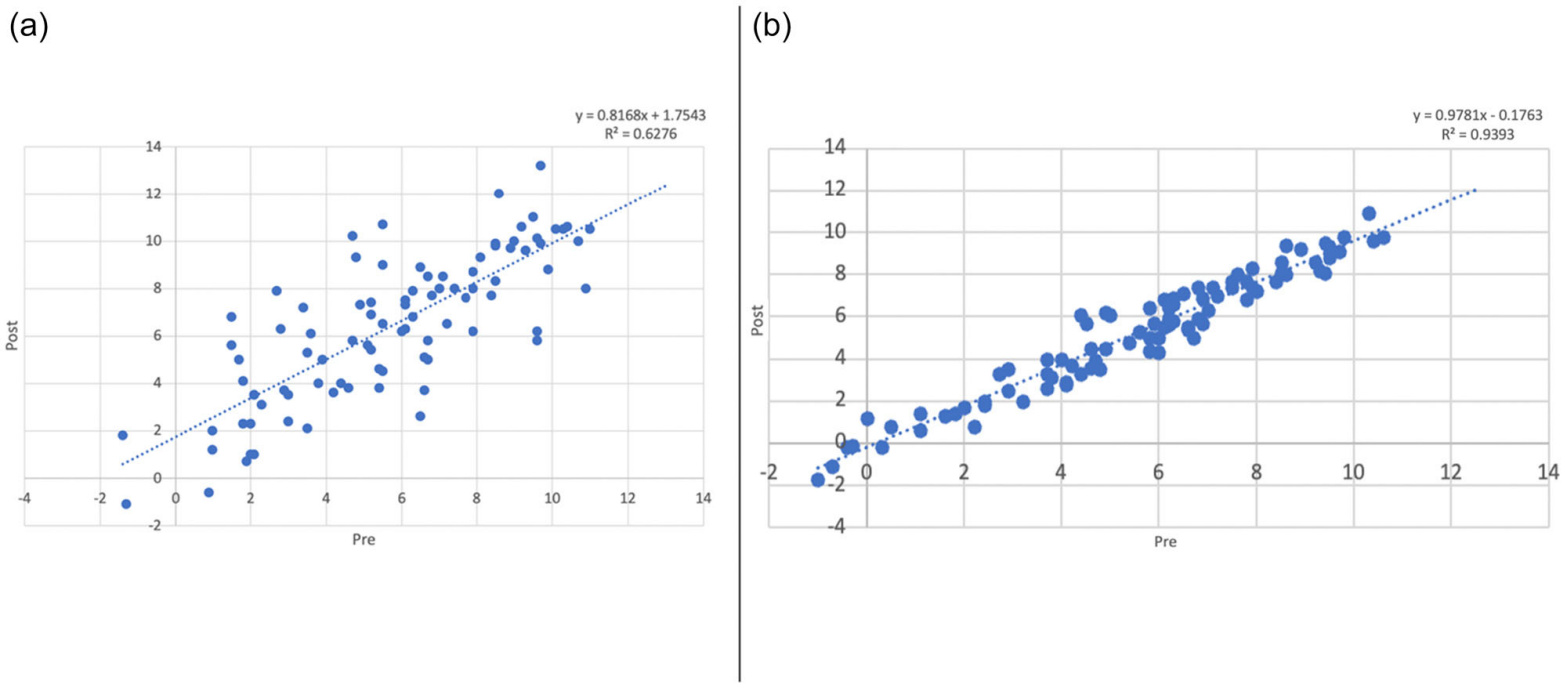
Scatter plot of preoperative and postoperative posterior tibial slope (PTS) in the control group (a) versus study group (b).

**Figure 4:**
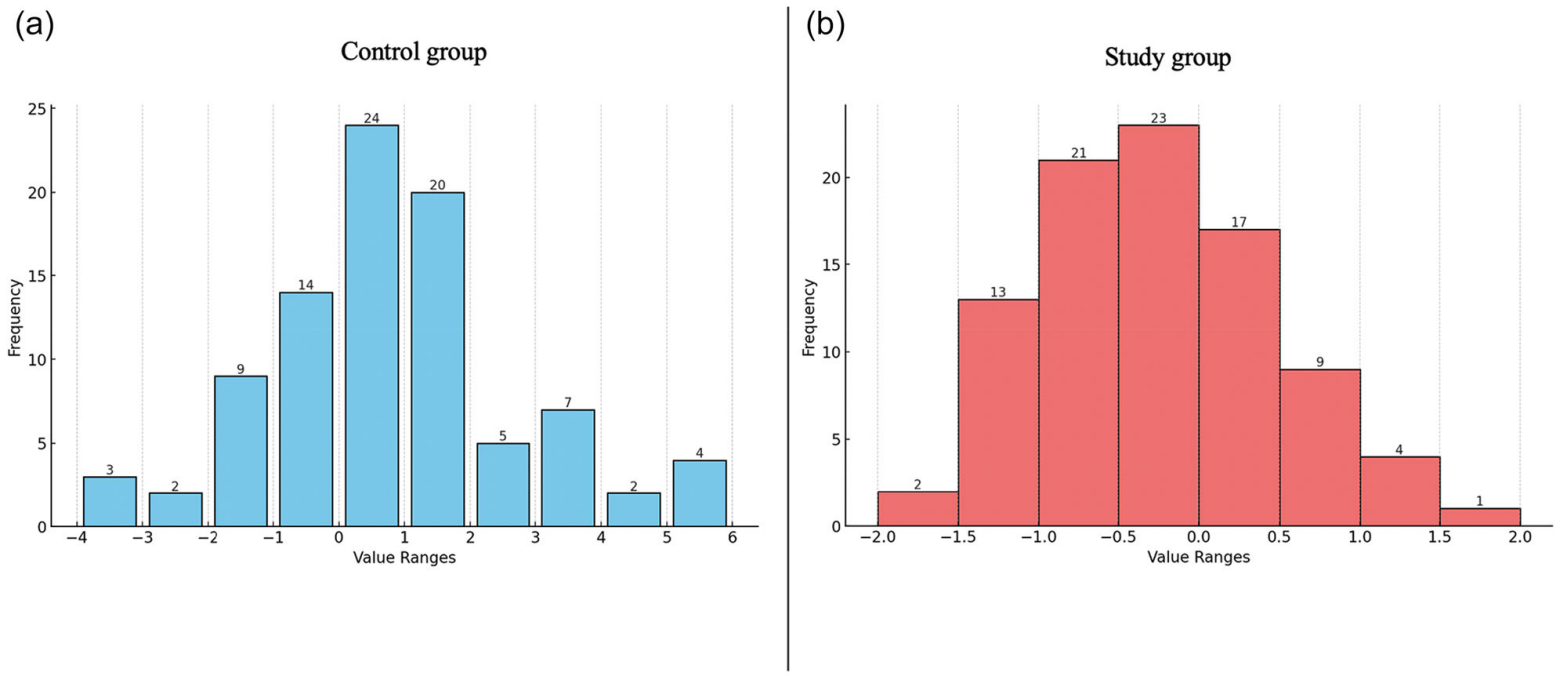
Values of posterior tibial slope difference (PTSD) in the control group (a) versus study group (b).

Patient demographics were not statistically different between the two groups (Table 1). The mean values of preoperative and postoperative PTS were 5.7° ± 3° and 6.4° ± 3.1°, respectively, in the control group, and 5.6° ± 2.9° and 5.3° ± 2.9°, respectively, in the study group (Table 2 and Figure 3). The mean value of PTSD was significantly lower in the study group compared to the control group (−0.3° ± 0.7° vs. 0.7° ± 2°; *p* < 0.001) with 0% and 25.6% outliers, respectively (Table 2 and Figure 4).

**Table 1:** Patients' demographics.

Demographics	Control group (*n* = 81)		Study group (*n* = 83)		*p*‐Value
	Mean (SD)	Range	Mean (SD)	Range	
Age	74.4 (8.8)	46–87	74.2 (8.5)	48 to 84	0.763
Gender (*n*)	39 M, 42 F	/	38 M, 45 F	/	0.762
Height	156.5 (9.1)	142–175	156.2 (8.9)	143–173	0.601
BMI	25.1 (3.7)	16.7–33.7	25.3 (3.4)	16.8–34.6	0.715
HKA	176.5 (6.6)	165.7–191.1	175.7 (6.3)	165.2–190.6	0.987
LDFA	88.5 (2.7)	85.7–93.5	88.2 (2.6)	85.3–92.9	0.361
MPTA	86.9 (2.9)	80.8–91.1	86.4 (2.7)	79.8–90.4	0.481

Abbreviations: BMI, body mass index; HKA, hip‐knee‐ankle angle; LDFA, lateral distal femoral angle; MPTA, medial proximal tibial angle; SD, standard deviation.

**Table 2:** Pre‐ and postoperative PTS, posterior tibial slope difference (PTSD), outliers and tibial recuts in the control group versus study group.

	Control group (*n* = 90)		Study group (*n* = 90)		*p*‐Value
	Mean (SD or %)	Range	Mean (SD or %)	Range	
Preoperative PTS	5.7° (3)	−1.4 to 11	5.6° (2.9)	−1 to 10.6	0.894
Postoperative PTS	6.4° (3.1)	−1.1 to 13.2	5.3° (2.9)	−1.7 to 10.9	0.019
PTSD	0.7° (2)	−3.9 to 5.5	−0.3° (0.7)	−1.7 to 1.7	<0.001
Outliers	23/90	25.6%	0/90	0%	<0.001
Recuts	14/90	15.6%	0/90	0%	<0.001

No statistically significant differences were observed in the PTSD and percentage of inliers and outliers between the cases performed by the lead surgeon and those performed by the senior resident in either group (Table 3). The assessment of the surgical reports revealed that a tibial recut to adjust the slope was performed in none of the cases in the study group and in 14/90 cases (15.6%) in the control group (*p* < 0.0001): 6/61 cases (9.8%) performed by the lead surgeon and 8/29 cases (27.6%) performed by the senior resident (*p* = 0.031) (Table 3).

**Table 3:** Posterior tibial slope difference and tibial recuts in the control and study group, divided by surgeon.

	Lead surgeon (*n* = 61)		Senior resident (*n* = 29)		*p*‐Value
	Value	%	Value	%	
PTSD					
Control group	0.7 ± 2.1	‐	0.7 ± 1.8	‐	0.89
Study group	−0.29 ± 0.7	‐	−0.31 ± 0.7	‐	0.93
Outliers					
Control group	16/61	26.2	7/29	24.1	0.833
Study group	0/62	0%	0/28	0%	1
Recuts					
Control group	6/61	9.8	8/29	27.6	0.031
Study group	0/62	0%	0/28	0%	1

Abbreviation: PTSD, posterior tibial slope difference.

## DISCUSSION

The main finding of this study was that the 'over‐the‐top' technique provides greater accuracy and reproducibility in setting the sagittal slope compared to the conventional KA approach—even when performed by less experienced surgeons.This is the first study to propose an alternative to the angel wing method of the original kinematic alignment technique.

The application of the ‘over‐the‐top’ technique resulted in a mean PTSD of −0.3° ± 0.7° (range: −1.7° to 1.7°), with 0% of outliers. On the other hand, the conventional KA technique resulted in a statistically significant higher PTSD (0.7° ± 2°; range: −3.9° to 5.5°), with 25.6% of outliers in the control group. These results are consistent with those reported by Nedopil et al. [26], who found a PTSD of 0.7° ± 3.2° using the conventional KA technique, with 75% of patients having a difference of less than 2°—a level of accuracy comparable to that achieved in robot‐assisted surgeries [3, 20, 30].

Similar techniques, employing a hook probe and a 2.5‐mm K‐wire inserted antero‐posteriorly on the medial tibial plateau, were recently described for unicompartmental knee arthroplasty (UKA) by Akagi et al. [1] and by Giurazza et al. [15], respectively. Akagi et al. [1] reported a mean implantation error in the sagittal plane of 1.1° ± 1.3°, with 73% of knees within 2° of the preoperative PTS. Likewise, Giurazza et al. [15] reported a mean sagittal slope difference of −0.7° ± 1.9° with 85.1% of knees within 3° of the preoperative PTS. The greater accuracy of the over‐the‐top technique likely stems from the complete exposure and direct visualisation of both the anterior and posterior borders of the medial tibial plateau, which is not achievable in UKA, for which these techniques were originally developed. Furthermore, while theoretically possible to apply these techniques in TKA, we argue that the over‐the‐top technique remains preferable for several reasons. First, it relies on the use of an angel wing—a tool universally available in all standard TKA instrument sets—ensuring broad applicability. Second, incorporating a hook probe or a K‐wire would introduce an extra step to the surgical procedure—finding parallelism between the probe/K‐wire and the saw blade—which could introduce additional sources of error.

Changes in posterior tibial slope greater than 2° and changes in polyethylene thickness greater than 1 mm [28] may significantly affect the range of internal‐external rotation achieved in the 0°–90° flexion arc. A reduction in internal tibial rotation during flexion, as a consequence of a slackening or tightening of the PCL, may decrease the range of motion [34], increase the risks of tibial component subsidence and polyethylene wear [19, 27], and impair the dynamic reduction of the Q‐angle during flexion, adversely affecting patellofemoral tracking and tension in the retinacular ligaments [9, 22, 23]. Therefore, if the surgeon can rely on the accuracy of the slope of the cut, the decision‐making process becomes simpler: knowing the slope is correct, the surgeon can focus solely on adjusting the insert thickness to optimise internal–external rotation, which should be in the 15°–18° range, as in the native knee [9, 10, 17].

The lack of a statistically significant difference in PTSD in the study group between cases performed by the lead surgeon and those by the senior resident, combined with the fact that no tibial recuts were required, underscores the excellent reproducibility of the over‐the‐top technique, even in the hands of less experienced surgeons—a common limitation associated with manual techniques [33]. In contrast, tibial recuts to adjust the slope were necessary in 15.6% of the cases in the control group, with a statistically significant difference between the lead surgeon and the senior resident (9.8% vs. 27.6%; *p* = 0.031).

One potential limitation of the described technique pertains to two specific anatomical scenarios that may impact the reliability of the posterior reference point: the presence of high tibial posterior osteophytes, and a subchondral depression often seen on the postero‐medial border of the medial tibial plateau in varus knees with ACL insufficiency and some femoro‐tibial subluxation. However, despite these challenges, direct visualisation of the entire medial tibial plateau enables the selection of an optimal posterior reference point, effectively excluding posterior osteophytes or subchondral depressions. Consequently, in all examined cases, their presence did not hinder the direct referencing method of the medial tibial plateau, even in significantly deformed knees. Nevertheless, in such cases, the ‘over‐the‐top’ technique offers an additional advantage over the conventional KA technique, as it requires less medial exposure, thereby preserving the integrity of the deep medial collateral ligament (MCL). This allows for a double check for parallelism with the deep MCL line, a useful anatomical landmark recently described by Parratte [31, 32], enhancing overall accuracy.

## LIMITATIONS

The present study has few limitations to be acknowledged. In many individuals, the medial and lateral posterior tibial slopes can differ by several degrees [24, 35]. Nevertheless, only the medial slope was assessed and reproduced in this study to maintain consistency with the original KA technique and because the internal tibial rotation guided by the PCL occurs around a fulcrum located in the medial compartment [9], replicated by the medial ball‐in‐socket implant used in this study [26].

Tibial slope was measured on plain radiographs, inherently less accurate compared to 3D imaging techniques such as EOS or CT scans [21, 25]. Nevertheless, strict exclusion criteria were applied to minimise issues related to poor tibial rotation and variations in tibial shaft length included in the image. Additionally, the selected method—based on the posterior tibial cortex—is recognised for its reliability, being the least affected by morphometric variables [4], and excellent intra‐observer and inter‐observer agreement were observed for all radiological measurements.

The short follow‐up period precluded the assessment of the over‐the‐top technique's impact on patient reported outcome measures (PROMs). While some studies report no significant differences in PROMs between slope reduction and preservation [2, 5, 34], the clinical implications of this aspect warrant further investigation. Nevertheless, the proposed technique can serve as a surgical reference even for those aiming to reduce the PTS. In fact, once the cutting guide is set to the native PTS value determined using our technique, further adjustments can be made based on this reference position.

## CONCLUSIONS

The ‘over‐the‐top’ technique is more accurate, reproducible and straightforward than the conventional KA technique in setting the posterior tibial slope. Its implementation may help even less experienced KA surgeons faithfully restore the native posterior tibial slope.

## AUTHOR CONTRIBUTIONS

Edoardo Franceschetti designed the described technique and was responsible for conceptualisation. Giancarlo Giurazza was responsible for writing of the manuscript and qualified as corresponding author. Pietro Gregori and Biagio Zampogna were responsible for data acquisition and realisation of Figures and Tables. Giuseppe Francesco Papalia and Stefano Campi were responsible for data analysis. Stephen M. Howell, Alexander J. Nedopil and Rocco Papalia were responsible for reviewing and critically revise the manuscript. All authors have given final approval of the version to be published.

## CONFLICTS OF INTEREST STATEMENT

The authors declare no conflicts of interest. Outside the current study S. M. Howell and A. J. Nedopil receives royalties from Medacta and are paid consultants for Medacta.

## ETHICS STATEMENT

The study was performed in accordance with the ethical standards as laid down in the 1964 Declaration of Helsinki and its later amendments. Institutional review board approval was obtained for this research (PAR 24.22 OSS). Written consent was obtained from all participants. No financial incentives were provided for participation.

## Data Availability

The data that support the findings of this study are available from the corresponding author, [G.G.], upon reasonable request.
